# Detection of Diabetic Eye Disease from Retinal Images Using a Deep Learning based CenterNet Model

**DOI:** 10.3390/s21165283

**Published:** 2021-08-05

**Authors:** Tahira Nazir, Marriam Nawaz, Junaid Rashid, Rabbia Mahum, Momina Masood, Awais Mehmood, Farooq Ali, Jungeun Kim, Hyuk-Yoon Kwon, Amir Hussain

**Affiliations:** 1 Department of Computer Science, University of Engineering and Technology Taxila, Taxila 47050, Pakistan; tahira.nazir@uettaxila.edu.pk (T.N.); marriam.nawaz@uettaxila.edu.pk (M.N.); rabbia.mahum@uettaxila.edu.pk (R.M.); momina.masood@uettaxila.edu.pk (M.M.); awais.mehmood@uettaxila.edu.pk (A.M.); farooq.ali@uettaxila.edu.pk (F.A.); 2 Department of Computer Science and Engineering, Kongju National University, Gongju 31080, Chungcheongnam-do, Korea; jekim@kongju.ac.kr; 3 Research Center for Electrical and Information Technology, Department of Industrial Engineering, Seoul National University of Science and Technology, Seoul 01811, Korea; 4 Centre of AI and Data Science, Edinburgh Napier University, Edinburgh EH11 4DY, UK; a.hussain@napier.ac.uk

**Keywords:** diabetic retinopathy, diabetic macular edema, medical imaging, deep learning, retinal images

## Abstract

Diabetic retinopathy (DR) is an eye disease that alters the blood vessels of a person suffering from diabetes. Diabetic macular edema (DME) occurs when DR affects the macula, which causes fluid accumulation in the macula. Efficient screening systems require experts to manually analyze images to recognize diseases. However, due to the challenging nature of the screening method and lack of trained human resources, devising effective screening-oriented treatment is an expensive task. Automated systems are trying to cope with these challenges; however, these methods do not generalize well to multiple diseases and real-world scenarios. To solve the aforementioned issues, we propose a new method comprising two main steps. The first involves dataset preparation and feature extraction and the other relates to improving a custom deep learning based CenterNet model trained for eye disease classification. Initially, we generate annotations for suspected samples to locate the precise region of interest, while the other part of the proposed solution trains the Center Net model over annotated images. Specifically, we use DenseNet-100 as a feature extraction method on which the one-stage detector, CenterNet, is employed to localize and classify the disease lesions. We evaluated our method over challenging datasets, namely, APTOS-2019 and IDRiD, and attained average accuracy of 97.93% and 98.10%, respectively. We also performed cross-dataset validation with benchmark EYEPACS and Diaretdb1 datasets. Both qualitative and quantitative results demonstrate that our proposed approach outperforms state-of-the-art methods due to more effective localization power of CenterNet, as it can easily recognize small lesions and deal with over-fitted training data. Our proposed framework is proficient in correctly locating and classifying disease lesions. In comparison to existing DR and DME classification approaches, our method can extract representative key points from low-intensity and noisy images and accurately classify them. Hence our approach can play an important role in automated detection and recognition of DR and DME lesions.

## 1. Introduction

Diabetes is a disorder in which the glucose level of patients is higher than the normal level. Diabetes victims are at risk of various eye diseases, such as diabetic retinopathy (DR), diabetic macular edema (DME), cataract, and glaucoma. DR is an eye disease that affects blood vessels of the retina of diabetic patients, and its signs include microaneurysms, hemorrhages, and soft and hard exudates. Microaneurysms are due to distortions in the boundary of blood vessels and appear as small red dots on the retina. Soft exudates are white lesions on the retina that occur due to occlusion of the arteriole, while hemorrhages are due to blood leakage from damaged vessels and appear as dark red spots. Hard exudates are yellow, brighter spots with a waxy appearance on the retina, which are formed because of the leakage of blood from vessels. DR is categorized into two main types, non-proliferative (NPDR) and proliferative DR (PDR) [1]; details are given in Table 1. NPDR is a common and early form of DR and is further divided into three types, which are mild, moderate and severe. The advanced level of DR is called PDR and is characterized by the development of abnormal fresh blood vessels in the retina; the new vessels may break out and leak into the retina causing vision loss. DME is the advanced stage of DR, which occurs when DR affects the macula region. DME results in the accretion of fluid in the macula area causing the swelling of the macula. The central region of the retina is the macula, which is responsible for our central and sharp vision [2,3].

According to the World Health Organization (WHO), as the prevalence of diabetes increases rapidly in the coming years, the proportion of those with DR is estimated to grow to 33% [4,5]. DR starts with minor abnormalities, in which vascular permeability increases. This can lead to PDR, in which new blood vessels are formed on the retina and later on the surface of the vitreous. However, DR is often undetected until it progresses to an advanced stage and leads to DME, which results in severe loss of vision. DME is a severe complication of DR and progresses when the retina gets thicker due to leaking blood vessels. According to a study conducted by the Early Treatment Diabetic Retinopathy Study (ETDRS) [6], the timely detection of DR and its level of severity can decrease the risk of blindness by 90%. Studies emphasized regular eye examinations for diabetic patients and suggested laser photocoagulation when needed to reduce visual complications. However, for the detection of DR, there are some implementation challenges, which include the need for human assessors, sociological and educational and financial sustainability [7,8]. Patients who have mild DR do not require any specific medical treatment, however they should ensure their diabetes is well controlled. They should be monitored periodically, to prevent the development of advanced stages of DR.

Techniques in the literature are unable to identify and classify DR and DME under intense light, color, size and angle variations. They also do not generalize well to unseen examples. Furthermore, existing studies are unable to accurately recognize early DR lesions due to their significantly small size. In this study, to cope with the challenges of existing work, we analyzed the risk of DR and DME by using Custom CenterNet. More specifically, we introduced DenseNet-100 as the backbone network for CenterNet to compute deep features from fundus samples. Retinal images can be significantly magnified to obtain the inner view of the eye and lesions related to DR and DME. The presented Custom CenterNet can detect all types of lesions, including early and severe signs of eye diseases. Below is the main contribution of our work:

1. A single method can detect the abnormalities or lesions of both diseases (DR and DME), which demonstrates the robustness of our method.
2. Efficient performance of proposed framework, due to improved CenterNet lesions detector.
3. Accurate localization of lesions, due to effective feature computation power of DenseNet-100 feature extractor.
4. Robust ability to detect the early stages of diseases, as CenterNet employs central pixel details.
5. Computationally effective framework, due to the simple and elegant architecture of CenterNet, as it employs a single-stage lesion detector.
6. We compared our method with the latest state-of-the-art techniques and achieved higher performance results.

The rest of the paper is organized as follows: Section 2 describes the proposed method; Section 3 consists of the experimental results and comparative analysis; Section 5 concludes our proposed work.

## 2. Related Work

Several machine learning (ML) approaches have been presented for DR and DME detection from retinal images. Seoud et al. [9] presented a method to automatically classify DR lesions by employing the concept of dynamic shape features (DSF). A multi-scale ring-shaped matched filter is applied to extract the features. The classification is performed using an RF classifier. The approach [9] exhibits superior lesion detection accuracy even under the presence of light and resolution variations in the input samples. However, the technique can only detect red lesions. Imani et al. [10] proposed a methodology to locate and classify DR lesions. Initially, the input image is pre-processed to obtain the green channel and to remove the unwanted artifacts from it by employing the Otsu thresholding algorithm. Then, the morphological component analysis (MCA) method is applied to compute the features from the images, which are later employed to train the support-vector machine (SVM) classifier. The technique [10] outperforms the challenging database; however, it may not perform well over the samples with post-processing operations. Zou et al. [11] introduced a method for DME detection from fundus images. Both feature and position properties are combined by employing SVM and the Bayesian probability theory to locate the DME from the input samples. The technique in [11] works well for DME-affected images; however, the performance requires further improvements. In [12] a hand-coded technique comprised of Dual-tree complex wavelet transform together with Gaussian data description (GDD) is employed for key points computation to determine various DME stages. The approach in [12] needs a performance improvement. Marin et al. [13] introduced a methodology for DME detection based on the Gaussian and difference of Gaussian filter bank. A regularized local regression (RLR) classifier is used to classify the possible DME regions. The approach in [13] is robust in DR screening; however, the performance needs to be further improved.

Deep learning (DL) techniques of artificial intelligence have been recognized as great innovations [14,15], in the medical field. These algorithms have been used in numerous areas, such as pathology [16], radiology [17] and dermatology, age-related macular degeneration (AMD) and premature retinopathy [18,19], etc. In ophthalmology, analysis of color fundus photographs (CFPs) has been used by DL algorithms for DR grading and heart disease risk factors [20]. DL models use representation learning methods to obtain meaningful patterns from the huge collection of retinal colored images. Li et al. [20] presented the latest dataset of fundus samples, namely, DDR, over which DL-based five categorization and five segmentation frameworks, namely, VGG-16, ResNet-18, DenseNet-121, SE-BN-Inception, DeepLab-v3+, HED, RCNN, YOLO and SSD, respectively, are evaluated. It is concluded, in [21], that DL-based approaches perform well for DME classification and segmentation; however, for small-size lesions, these techniques require further improvement. Perdomo et al. [22] proposed a framework for DR screening by employing a two-stage CNN architecture. Initially, deep features are computed from the input sample through an eight-layered CNN framework, based on which AlexNet architecture is trained to classify the healthy and DME-affected images. The approach in [22] works well for DME classification; however, it requires a huge training set. Abramoff et al. [23] introduced a DL approach for the automated identification of DR. The approach in [23] reported a better performance, compared to simple hand-coded-based techniques. Gargeya et al. [24] proposed a fully automated framework by employing the DL approach for DR recognition. The work in [24] explained the possible application of DL frameworks in DR screening. Tan et al. [25] proposed a 10-layer CNN framework to both segment and differentiate exudates, hemorrhages and microaneurysms. Although extensive research work has been presented for DR screening, how these approaches work in real-world scenarios needs to be explored. Moreover, existing literature on DR and DME detection is unable to achieve better classification performance over complex databases, as fundus samples change greatly because of varying fundus cameras, acquisition settings and DR victims. Therefore, there is still a need to develop a more effective and efficient DR and DME screening method.

## 3. Proposed Methodology

**Figure 1:**
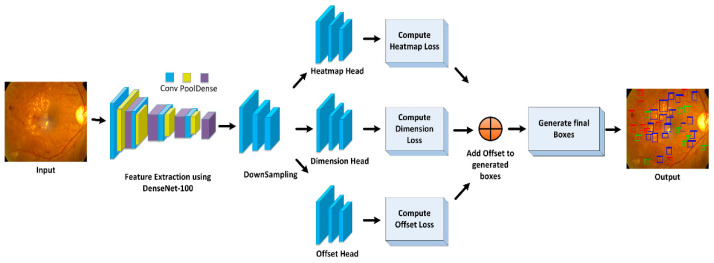
Flow diagram of proposed technique.

The proposed method is comprised of two main phases, i.e., transfer learning and localization and classification. The complete functionality is shown in Figure 1.

The presented work comprises two main parts. The first is ‘dataset preparation’ and the other is an improved CenterNet network trained for eye disease classification. Firstly, we develop annotations for images with disease, to specify the exact region of interest, while the other part of the established framework trains CenterNet [26] over the annotated samples. We employed Custom CenterNet with DenseNet-100 as its base network for feature computation. The features extractor of the Custom CenterNet framework, namely, DenseNet-100, accepts image sample and location of the affected region in the input image. Figure 1 illustrates the architecture of the presented method. In the beginning, an input sample, along with the bounding box (bbox), is passed to the DenseNet-100 framework. The bbox recognizes the region of interest (RoI) in CNN key points. Following this, the Custom CenterNet is trained to classify the located areas. Finally, accuracies are estimated for all units as per metrics being utilized in the area of computer vision.

### 3.1. Annotations

To ensure an efficient training process, it is mandatory to accurately specify the position of the affected region from the input retinal samples. For this purpose, we utilized the LabelImg [27] software to build the sample annotations. The generated annotations are stored in an XML file which comprises two important details: (i) class associated with each affected region and (ii) box values for drawing a rectangular box over the detected region. In the next step, a train. record file is produced from an XML file, which is utilized for training the model.

### 3.2. CenterNet

Efficient key-points computation is needed to precisely categorize eye diseases into numerous classes. However, computing a discriminative set of the feature vector is a challenging task because of the following reasons: (i) models can result in over-fitting by employing the large size key-points vectors; (ii) while utilizing a small key-points set, the technique may miss learning some important object behaviors, i.e., texture and color changes, which make affected regions of disease indistinguishable from the healthy portion.

To achieve the discriminative and robust set of features, it is mandatory to employ an automated features computation technique without the need of using hand-crafted features calculation. The models using handcrafted key points are not robust in correctly identifying and recognizing eye diseases because of extensive changes in the size, texture, color and position of lesions. To deal with the challenges, we employed a DL-based framework, namely, Custom CenterNet, because of its ability to directly compute the efficient features from the input samples. The convolution filters of CenterNet calculate the key points of the suspected image by analyzing its structure. The motivation for using CenterNet [26] over the RCNN, Fast-RCNN [28] and Faster-RCNN [15,29] approaches for eye disease recognition is that these approaches perform classification by following a two-stage object detector. In [29], initially, a Region Proposal Network (RPN) is employed to locate the regions of interest (RoIs), which possibly surround an object. Then, using the collective key points intimate with each RoIs, separated identification heads of the framework detect the category of object and draw the rectangular box. Therefore, these methods are computationally inefficient, as they are not robust for real-time object detection requirements. CenterNet better addresses the limitations of RCNN, Fast-RCNN and Faster-RCNN by specifying both features and location boxes of objects in input samples at the same time. Therefore, the one-stage object detection power of CenterNet makes it computationally efficient and better at generalizing real-time object detection.

For eye disease classification, it is a complex task to identify the key points of interest due to the following reasons: (i) locating the actual position of the affected region from the input sample, due to intense light and color variations, and (ii) determining the class associated with each object. The CenterNet technique can efficiently detect and classify affected regions of different classes by employing its heat-maps and by replacing the two-stage object detection with a one-stage recognition algorithm. The heat-map module works by employing the center of key points and achieves higher recall rates, which helps to reduce the features computation cost of the proposed framework.

### 3.3. Custom CenterNet

**Figure 2:**
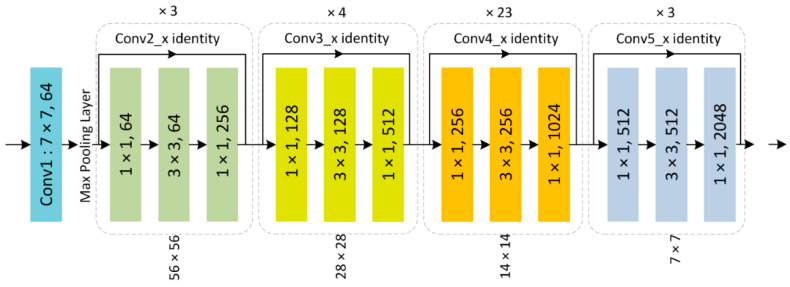
ResNet-101 Architecture.

The traditional architecture of CenterNet [30] employed ResNet to compute image key points and conduct image medical analysis. However, the ResNet framework utilizes skip-connections and employs identity methods to avoid non-linear transformations, which cause the direct flow of gradient from the back layers to the front ones by using the identity function. Figure 2 shows the structural description of the ResNet-101 framework. The ResNet-101 model comprises huge parameters, which eventually causes the vanishing gradient problem. To cope with the problem of the ResNet-101 framework, we present a densely associated convolution framework, namely, DenseNet, as the base network of the conventional CenterNet technique, by substituting ResNet-101 with DenseNet-100. The presented feature extractor, namely, DenseNet-100, has fewer parameters and has a thinner layer network, in comparison to ResNet-101, which makes it more cost-efficient. DenseNet contains several dense blocks (DBs) successively joined to each other through additional convolutional and pooling layers between consecutive DBs. The DenseNet framework can show the complicated transformation which helps overcome the problem of the deficiency of the resultant location information for the top-level features, to some extent. Furthermore, DenseNet supports the feature propagation procedure and boosts their reusability, which makes them highly convenient for DR and DME recognition and fastens the training process. Therefore, in the proposed solution, DenseNet-100 [31] is employed as a key-points calculator for CenterNet.

The Algorithm 1 shows the steps for CenterNet model.
**Algorithm 1** Steps to train the Custom CenterNet modelINPUT: TS, annotations OUTPUT: Localized RoI, CM, Classified lesion        *TS*–training set.        annotations–Position of DR and DME lesions in retinal images        Localized RoI–lesion location in the output        *CM*–CenterNet model with DenseNet-100 backbone.        Classified lesion–Class associated with each detected lesion. imageSize ← [x y] // Approximation of Bounding box    *α* ← AnchorsEstimation (*TS*, annotation) // CenterNet Model    *CM* ← DenseNet100Based CenterNet (*imageSize*, *α*)    [*I_t_*, *I_s_*] ← division of samples into *training* and *testing dataset* // Training Unit of lesions Identification     For each image *i* from → *I_t_*        Compute *DenseNet100* keypoints → ts    End For    Training *CM* over ts, and calculate training time *t_dense*    *η_ dense* ← PreLesionPosition(ts)    *Ap_ dense* ← Evaluate_AP (*DenseNet-100*, *η_ dense*)    For each sample *I* from → *I_s_*          (a) extract features through trained framework € → β*I*          (b) [*bounding_box*, *objectness_ score*, *output label*] ← Predict (β*I*)          (c) show sample along with *bounding_box*, *output label*          (d) *η* ← [*η bounding_box*]    End For *Ap*_€ ← Evaluate framework € using *η* *Output_class* ← CM (*Ap*_€).

#### 3.3.1. Feature Extraction Using DenseNet-100

DenseNet-100 comprises four densely associated modules with the same number of layers as ResNet-101. However, the DenseNet-100 framework contains fewer parameters, which gives it a computational advantage over the ResNet-101 model. The structural details of the DenseNet-100 are demonstrated in Table 2.

**Figure 3:**
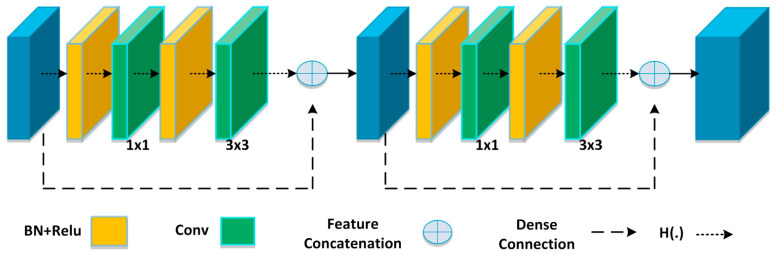
DenseNet–architecture.

The DB is the essential element of DenseNet-100, as presented in Figure 3, in which N × N × M0 exhibits the features maps (FMs) of the *n*-1 layer. The size of the FMs is N, while the total channels are represented with M0. A non-linear transformation H(.)comprises several methods, for example, a batch normalization layer (BN), a Rectified Linear Unit (ReLU) activation function, a 1 × 1 convolution layer (ConvL), employed to minimize the total channels, and a 3 × 3 ConvL, utilized for key-points rearrangement. The long-dashed arrows are used to show the dense links, which are employed to combine the *n*-1 layer to the *n* layer and perform concatenation by using the output of the H(.). In the end, N × N × (M0 + 2M) is the outcome of the n + 1 layer.

The extensive dense links increase FMs; therefore, the transition layer is introduced to reduce the key-points dimension from the previous DB, as discussed in [32,33]. The computed features from DenseNet-100 are down-sampled with the stride rate R = 4 and are then passed to compute three types of heads, described in the following sections.

#### 3.3.2. Heatmap Head

Heatmap head presents a features estimation over the down-sampled deep features from the DenseNet-100 framework to locate the affected regions of disease together with the respective class. Key points are bbox center in case of object detection, which is computed by using Equation (1):

(1) O^i,j,c=exp(−(i−p^i)2+(j−p^j)22σp2)

where *i* and *j* are the actual key point coordinates, ${\hat{p}}_{i}$ and ${\hat{p}}_{j}$ are the locations of predicted down-sampled key points, *σ_p_* shows the object size-adaptive standard deviation, *c* is the number of classes and ${\hat{O}}_{x,y,c}$ represents the center for a candidate key points, if it has a value of one; otherwise, it is marked as background.

#### 3.3.3. Dimension Head

The dimension head is responsible for predicting the coordinates of the box. The dimension of the bbox for a candidate object *k* with class *c* having coordinates (*x*_1_, *x*_2_, *y*_1_, *y*_2_) can be estimated through the L1 norm, which is (*x*_2_–*x*_1_, *y*_2_–*y*_1_).

#### 3.3.4. Offset Head

The offset head is computed to minimize the discretization error which occurs because of performing down-sampling over the input sample. After computing the center points, these points are again mapped to a higher dimensional input image.

#### 3.3.5. Multitask Loss

CenterNet is an end-to-end learning technique that employs multi-task loss methods to improve its performance and accurately localize the affected region with the corresponding class. For this purpose, the proposed model uses a multi-task loss *L* on each sampled head, defined as in Equation (2):

(2) $$L_{centernet}=L_{map}+\lambda_{dim}L_{dim}+\lambda_{off}L_{off}$$

where $L_{CenterNet}$ is the total loss computed by CenterNet, $L_{map}$, $L_{dim}$ and $L_{off}$ are the heatmap, dimension and offset head losses, respectively. $\lambda_{dim}$ and $\lambda_{off}$ are constants with the values of 0.1 and 1, respectively.

The heatmap loss $L_{map}$ is computed using Equation (3), as follows:

(3) $$L_{map}=\frac{-1}{n}\sum_{i,j,c}\left\{ \begin{array}{l} {\left(1-{\hat{O}}_{i,j,c}\right)}^{\alpha }\log({\hat{O}}_{i,j,c})\;if\;{\hat{O}}_{i,j,c}=1\\ {\left(1-O_{i,j,c}\right)}^{\beta }{\left({\hat{O}}_{i,j,c}\right)}^{\alpha }\log(1-{\hat{O}}_{i,j,c})\;otherwise \end{array}\right.$$

where *n* is the total number of key points, $O_{i,j,c}$ is the actual candidate key point center, and ${\hat{O}}_{i,j,c}$ is the predicted key point center. *α* and *β* are the hyperparameters of heatmap loss with the values of 2 and 4 for all our experiments, respectively.

The dimension head loss is calculated using Equation (4):

(4) $$L_{dim}=\frac{1}{n}\sum_{k=1}^{n}\left|{\hat{b}}_{k}-b_{k}\right|$$

where ${\hat{b}}_{k}$ is the predicted bbox coordinates, $b_{k}$ is the actual dimensions of bboxes from ground truths and *n* is the total number of the samples.

Finally, the offset-head loss is calculated with Equation (5):

(5) Loff=1n∑p|F^p^−(pR−p^)|

where ${\hat{F}}_{\;}$ is the predicted offset value, p is actual and $\hat{p}$ is the down-sampled key point and *R* is the output stride.

#### 3.3.6. Bounding Box Estimation

At the inference stage, the computed peaks against each class from the heatmaps are taken independently. In our case, the responses having a value larger or equal to its 8-connected neighbors are considered and the top 100 peaks are selected. Suppose $\hat{Q}$ is presenting *N* identified center points of category *c* given by Equation (6):

(6) $$\hat{Q}={\left\{\left({\hat{x}}_{j},{\hat{y}}_{j}\right)\right\}}_{j=1}^{N}$$

where (${\hat{x}}_{j},{\hat{y}}_{j}$) is an integer coordinate, showing the position of each detected keypoint. In our method, we employ all keypoints values (represented by ${\hat{O}}_{x,y,c}$) as the computation of its identification confidence and the final bounding box is generated by using Equation (7):

(7) $$\begin{array}{c} ({\hat{x}}_{j}+{\hat{\partial x}}_{j}-{\hat{w}}_{j}/2,\;{\hat{y}}_{j}+{\hat{\partial y}}_{j}-{\hat{h}}_{j}/2,\\ {\hat{x}}_{j}+{\hat{\partial x}}_{j}+{\hat{w}}_{j}/2,\;{\hat{y}}_{j}+{\hat{\partial y}}_{j}+{\hat{h}}_{j}/2) \end{array}$$

where $\left({\hat{\partial x}}_{j},{\hat{\partial y}}_{j}\right)$ is the offset prediction denoted by ${\hat{O}}_{\hat{x},\hat{y}}$, while (${\hat{w}}_{j},\;{\hat{w}}_{j})$ is the size prediction denoted by ${\hat{d}}_{\hat{x},\hat{y}}$. The resultant bounding box is generated directly from the estimation of the features without employing IoU-based non-maxima suppression (NMS).

### 3.4. Detection Process

CenterNet is a DL-based framework that is independent of the approaches, such as selective search and proposal creation. Therefore, the suspected image, along with the bbox, is passed as input to the trained model, of which CenterNet calculates the center points of the eye diseases portion, the offsets to the x and y coordinates and the dimensions of bboxes along with the associated class. In our work, the hyper-parameters employed for model training are Epochs, which are set to 150 and 0.001 value of learning rate.

## 4. Performance Evaluation

In this section, we detail the database evaluation metrics utilized in our experiments. We describe the experimental results and comparative analysis with other techniques.

### 4.1. Dataset

For performance assessment of the introduced technique, we used two different datasets, i.e., APTOS 2019 and IDRiD.

**Figure 4:**
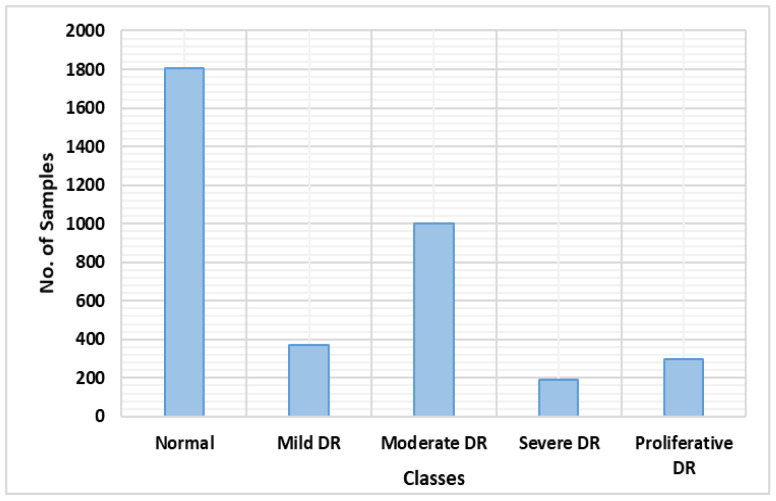
Class-wise distribution of the APTOS-2019 dataset.

To evaluate the DR and DME detection and classification performance of our proposed methodology, we employed the two publicly available datasets, namely, APTOS-2019 [34] and Indian Diabetic Retinopathy Image Dataset (IDRiD) [35]. The first dataset, namely, APTOS-2019, is provided by the Asia Pacific Tele-Ophthalmology Society in the Kaggle challenge. This database is comprised of a total of 3662 retinal samples, which are categorized into five classes, namely, negative, mild, moderate, proliferative and severe DR, as shown in Figure 4. For the first class, namely, negative DR, there are 1805 samples; for the other classes, namely, mild, moderate, severe and proliferative DR, there are 370, 999, 193 and 295 images, respectively. The IDRiD dataset consists of a total of 516 images, in which 413 samples are from the training set and the remaining 103 are from the testing set. Each sample has a grading ground-truth for two types of eye abnormalities, namely, DR (5 classes) and DME (3 classes).

### 4.2. Evaluation Metrics

We evaluated our proposed technique using different evaluation metrics, e.g., Intersection over Union (IOU), accuracy, precision, recall and mean average precision (mAP).

(8) $$\mathrm{Accuracy}=\frac{\mathrm{TP}+\mathrm{TN}}{\mathrm{TP}+\mathrm{FP}+\mathrm{TN}+\mathrm{FN}}$$

where, TP, TN, FP and FN show the true positive, true negative, false positive and false negative, respectively.

Equation (9) shows the mAP, in which AP denotes the average precision of each class and *q* is the query or test image. *Q* is the total number of test images:

(9) $$DSC=mAP∶=\sum_{i=1}^{Q}\frac{AP(q_{i})}{Q}$$

### 4.3. Results

We evaluated the detection performance of different DNN-based object identification frameworks for eye disease classification. We checked the recognition power of these approaches for various cases, i.e., for the occurrence of numerous lesions in a single sample or for the moles of various classes (DR and DME) to analyze whether these networks can identify the healthy and affected eye regions with complex background settings.

To accomplish this, we considered two types of object detection models, namely, one-stage (CornerNet) and two-stage detectors (Faster-RCNN and Mask-RCNN). The key difference between both models is that two-stage detectors work by first locating positions of the primary object in an image via employing several region proposal techniques, which are later narrowed down; then, the final classification task is performed. While in the case of single-stage detectors, both the class and location boxes of primary objects in input samples are defined in a single step.

To conduct the performance analysis of all object-detection models, we used the mAP metric, as it was selected by many researchers as a standard metric in object identification problems. Moreover, we compared the test time of all the models to analyze them in the aspect of computational complexity. From the reported results, it can be seen that the presented framework has attained the highest mAP value with minimum test time. The Mask-RCNN with ResNet-101 has attained comparable results with the presented technique; however, it is computationally more expensive due to its two-stage detector network. Moreover, in the case of single-stage detectors, the Corner model is unable to locate lesions of small sizes. The presented technique better addresses the limitations of existing one-stage and two-stage detectors by introducing the custom CenterNet with DenseNet-100 base network model. The DenseNet allows CenterNet to learn the more representative set of features, which assists in better locating the eye lesions of various categories. Moreover, the one-stage detector nature of CenterNet gives it a computational advantage over other models. The comparative results are presented in Table 3, which show that our proposed method achieves 96% mAP which is higher than the other methods.

#### 4.3.1. Localization of Disease Lesions

The correct detection of eye diseases is important to build an effective model for the automated recognition of eye lesions. For this purpose, we investigated the localization power of the presented technique by using an experiment. To quantitatively measure the localization power of the presented technique, we employed two metrics, namely, mAP and IOU. These metrics help to analyze how well the system can recognize eye lesions of several types. To localize the lesions of DR and DME from retinal images, lesions are considered as positive while the rest of the regions, including background, are considered as negative. The overlapped portion is marked using threshold IOU and greater than 0.7 regions are marked as affected. When the IOU value is less than 0.3, the regions are included in the background.

**Figure 5:**
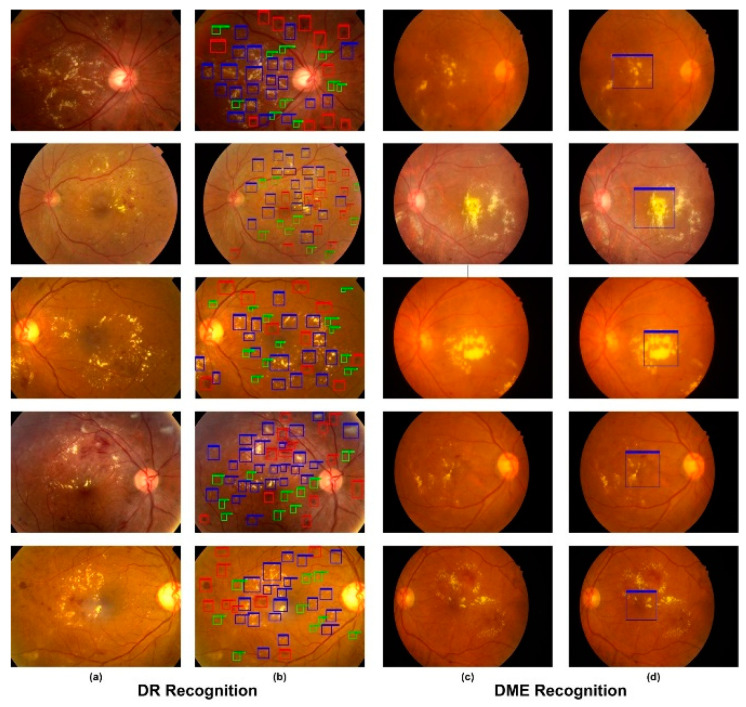
Test results of the proposed method. (**a**) Input images. (**b**) Localization results of DR regions; blue color shows hard exudates, green color shows microaneurysms and red color shows hemorrhages. (**c**) Input images of the IDRiD dataset. (**d**) Localization results of DME, which shows the macula region.

In our experiments, we utilized the Custom CenterNet approach for the localization of DR and DME lesions from retinal images. In the case of DR, we recognized the four types of lesions, i.e., hemorrhages, microaneurysms, hard and soft exudates, while, in the case of DME, only the macula region was localized. The localization results of both diseases are shown in Figure 5, showing lesion location, class and confidence score for each region which was localized by the proposed method. More precisely, we attained the mAP of 0.96 and 0.965 and the IoU of 0.973 and 0.975 for DR and DME lesions, respectively. Both the qualitative and quantitative results show that the presented technique can be reliably employed to localize and classify eye diseases.

#### 4.3.2. Classification

**Figure 6:**
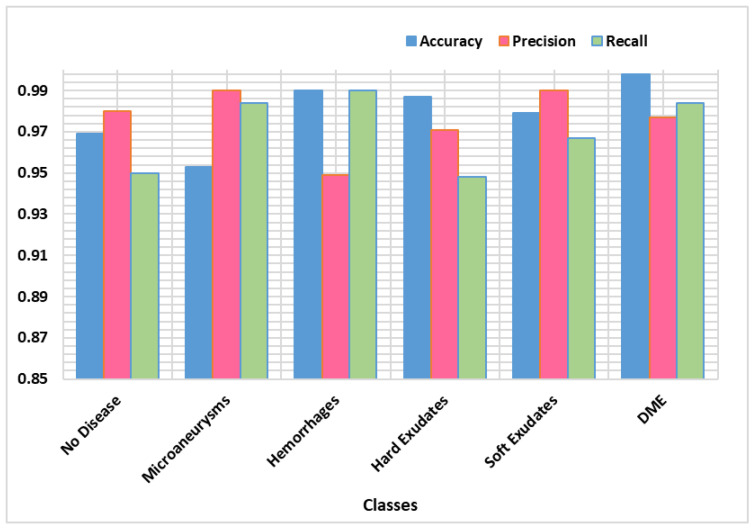
Stage-wise performance of our proposed technique.

**Figure 7:**
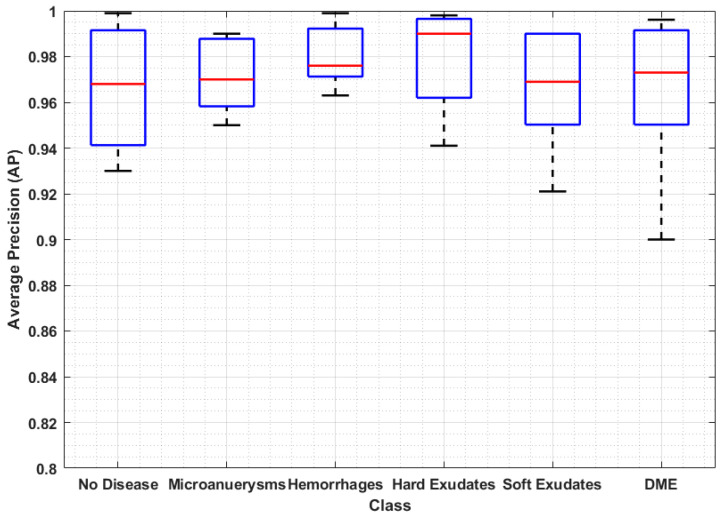
Average precision results of the proposed method.

The ability of the presented technique in identifying the severity of eye disease was also evaluated in our experiments. The trained Custom CenterNet model is utilized over the input test samples of both datasets for the classification of DR and DME. The stage-wise results are reported in Figure 6. In terms of precision, recall and accuracy, according to the results, we can say that method has achieved remarkable performance for classification and recognition of DR and DME from retinal images. Moreover, our prosed technique can efficiently find the severity level of eye disease because of accurate feature extraction using DenseNet-100. Our method precisely detects the DR and DME disease along with the early stages of DR, which was a challenging task, due to their small size. The AP results are plotted in the boxplot graph (Figure 7), as a boxplot can better demonstrate the obtained output by exhibiting the maximum, minimum and median of the AP achieved over the five categories of lesions.

**Figure 8:**
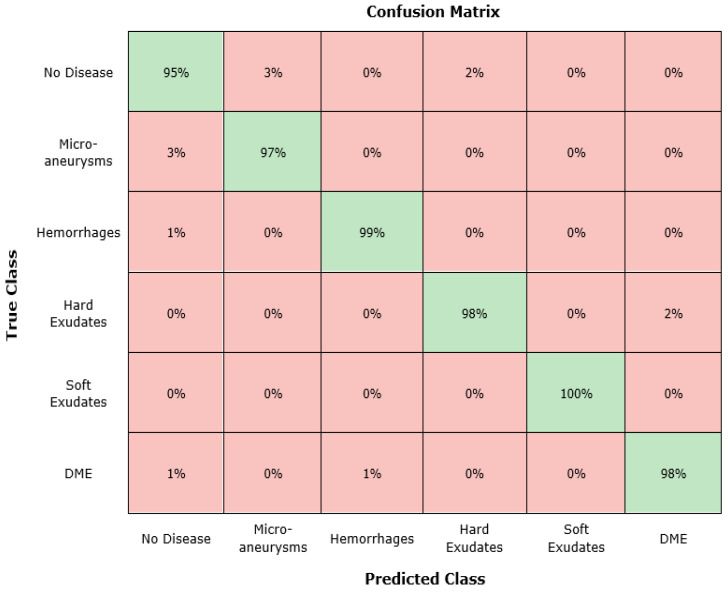
Confusion matrix.

Furthermore, to show the class-wise categorization power of the presented technique, we report the confusion matrix, which can assist in showing how well the Custom-CenterNet has predicted the actual class (Figure 8). It can be seen, from the confusion matrix, that Custom CenterNet attains the best classification performance for class and class having a positive rate (TPR) of 97%, whereas the proposed technique exhibits lower results for class (no disease) because of its textural similarity with class (microaneurysms). From Figure 8, it can be seen that the presented approach shows an average FPR of 2.16%, which shows the robustness of our framework. While, for the rest of the classes, the presented method also shows remarkable performance.

#### 4.3.3. Comparative Analysis

We implemented the proposed method in Python TensorFlow on a GPU-based machine. We compared our method results with other techniques on both datasets, namely, APTOS-2019 and IDRiD. For the APTOS-2019 database, we considered the reported results of the comparative techniques, i.e., Bodapati et al. [36], Chaturvedi et al. [37] and Bodapati et al. [38], and the results are reported in Table 4. While, for the IDRiD dataset, we compared our results with the approaches of Wu et al. [39] and Luo et al. [40] and an accuracy comparison is demonstrated in Table 5.

The reported results show that our method achieved better performance than the comparative approaches, in terms of accuracy. It can be seen that our method achieved an average accuracy of 97.93% on the APTOS-2019 dataset and 98.10% on the IDRiD dataset, which is higher than other methods. The comparative methods achieved an average accuracy of 92.74 on the APTOS-2019 dataset and 73.98 on the IDRiD dataset; therefore, we can say that our methods achieved a 5.19% and 24% performance gain on the APTOS-2019 and IDRiD datasets, respectively. The approaches are suffering from high computational cost and may not locate the lesions of varying sizes under intense light and color variations. However, the presented framework provides an efficient set of features that assists in locating the lesions even under the presence of post-processing attacks, i.e., blurring, noise, light, color and size variations. Moreover, the proposed method gives a computational advantage over the comparative methods due to its single-stage object detector network. Therefore, it can be concluded that the presented approach is both efficient and effective for eye disease classification.

#### 4.3.4. Cross-Dataset Validation

**Figure 9:**
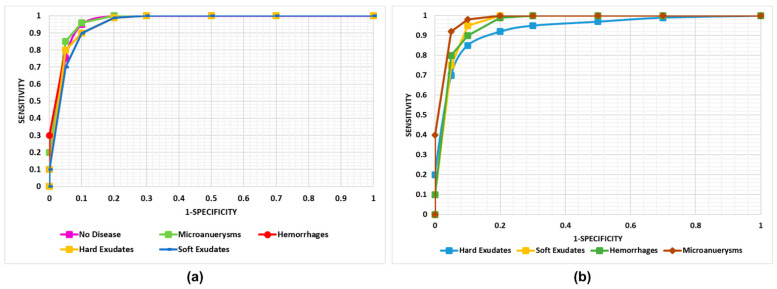
Cross dataset performance of our technique. (**a**) Test over the EYEPACS dataset. (**b**) Test over the Diaretdb1 dataset.

We experimented to check the identification performance of our framework over a cross-database scenario. The basic reason for executing cross-database validation is to check the generalization performance of the presented method. To accomplish this, we trained our technique on the APTOS-2019 and IDRiD datasets and tested it over the EYEPACS [41] and Darietdb1 [42] databases. EYEPACS is a challenging dataset that contains a total of 88,704 samples with five classes of lesions. The results are shown in Figure 9, which contains the class-wise test results of the EYEPACS and Diaretdb1 datasets. We plotted the results in terms of ROC curves. Figure 9a describes the test results of five classes over the EYEPACS dataset in terms of AUC, while Figure 9b shows the AUC values of the Diaretdb1 dataset having 4 classes or lesions. From these experiments or class-wise performance of both datasets, we can say that the presented work is robust in lesion detection from unseen images. Hence, it can be said that the introduced approach is robust in DR and DME identification and categorization.

## 5. Conclusions

The manual localization of DR and DME lesions requires experienced human experts to locate finer points of interest from colored fundus images, and classify them into appropriate groups through a grading system. To cope with the challenges of a manual detection system, a robust automated technique based on a custom CenterNet model and a DenseNet-100 feature extractor is introduced in the proposed work. We evaluated our approach on two benchmark datasets, namely, APTOS-2019 and IDRiD, and achieved accuracies of 97.93% and 98.10%, respectively. Our reported results demonstrate the capability of our method for accurately detecting and classifying both DR and DME lesions. In comparison to existing DR and DME detection techniques, our proposed framework can compute representative key points from low-intensity and noisy data images and accurately classify them to their respective classes. Hence, our approach can play an important role in the automated detection and recognition of DR and DME lesions. In future, we plan to evaluate our technique on more challenging datasets and adapt it for the detection of other diseases.

## Figures and Tables

**Table 1:** DR stages description.

Severity Level	Description
No DR	No abnormality
Mild NPDR	Microaneurysms are present
Moderate NPDR	Microaneurysms, hemorrhages, hard exudates are present
Severe NPDR	Observable beading in 2 or more quadrants/intra-retinal microvascular abnormality (IRMA) in 5/more quadrants/intra-retinal hemorrhages (more than 20) in each of the 4 quadrants
PDR	Neo-vascularization or vitreous hemorrhages

**Table 2:** Details of DenseNet-100.

	Of Maps	Layer
First layer	32×32×24	3×3 conv
DB 1	32×32×216	$\left(\begin{array}{l} 1\times 1\;conv\\ 3\times 3\;conv \end{array}\right)\times 16$
Transition layer 1	32×32×108 16×16×108	1×1 conv 2×2 ave_pool
DB 2	16×16×300	$\left(\begin{array}{l} 1\times 1\;conv\\ 3\times 3\;conv \end{array}\right)\times 16$
Transition layer 2	16×16×150 8×8×150	1×1 conv 2×2 ave_pool
DB 3	8×8×342	$\left(\begin{array}{l} 1\times 1\;conv\\ 3\times 3\;conv \end{array}\right)\times 16$
Final layer	1×342 1×10	8×8 ave_pool fully_connected

**Table 3:** Performance comparison of our technique with other RCNN approaches.

Technique	mAP	IoU	Time (s)
Faster RCNN	0.942	0.939	0.25
Mask RCNN	0.910	0.920	0.23
CornerNet	0.956	0.951	0.23
Proposed	0.970	0.974	0.21

**Table 4:** Comparison with state-of-the-art approaches over the Aptos-2019 dataset.

Method	Accuracy (%)
Bodapati et.al [36]	97.41
Chaturvedi et al. [37]	96.51
Bodapati et al. [38]	84.31
Proposed	97.93

**Table 5:** Comparison with state-of-the-art approaches over the IDRiD dataset.

Method	Accuracy (%)
Wu et al. [39]	80.00
Luo et al. [40]	67.96
Proposed	98.10

## Data Availability

Data sharing not applicable to this article as authors have used publicly available datasets, whose details are included in the “experimental results and discussions” section of this article. Please contact the authors for further requests.
